# Pulmonary arteries in coelacanths shed light on the vasculature evolution of air-breathing organs in vertebrates

**DOI:** 10.1038/s41598-024-61065-8

**Published:** 2024-05-09

**Authors:** Camila Cupello, Gaël Clément, Marc Herbin, François J. Meunier, Paulo M. Brito

**Affiliations:** 1 Departamento de Zoologia, Instituto de Biologia-IBRAG, Universidade do Estado do Rio de Janeiro, Rio de Janeiro, RJ Brazil; 2 Département Origines & Evolution, Muséum national d’Histoire naturelle, UMR 7207 (MNHN–CNRS–Sorbonne Universités) Centre de Recherche en Paléontologie (CR2P), Paris, France; 3 Département Adaptations du Vivant, Muséum national d’Histoire naturelle, UMR 7179 (CNRS-MNHN) Mécanismes Adaptatifs et Evolution (MECADEV), Paris, France; 4 Département Adaptations du Vivant, Muséum national d’Histoire naturelle, UMR 8067 (CNRS-IRD-MNHN-Sorbonne Universités-UCN, UA), Laboratoire de Biologie des Organismes et des Ecosystèmes Aquatiques (BOREA), Paris, France

**Keywords:** Palaeontology, Ichthyology

## Abstract

To date, the presence of pulmonary organs in the fossil record is extremely rare. Among extant vertebrates, lungs are described in actinopterygian polypterids and in all sarcopterygians, including coelacanths and lungfish. However, vasculature of pulmonary arteries has never been accurately identified neither in fossil nor extant coelacanths due to the paucity of fossil preservation of pulmonary organs and limitations of invasive studies in extant specimens. Here we present the first description of the pulmonary vasculature in both fossil and extant actinistian, a non-tetrapod sarcopterygian clade, contributing to a more in-depth discussion on the morphology of these structures and on the possible homology between vertebrate air-filled organs (lungs of sarcopterygians, lungs of actinopterygians, and gas bladders of actinopterygians).

## Introduction

Vascular canals and/or spaces are rarely documented in fossil bones. They have been described using both traditional techniques, such as ground sections (e.g. the Devonian osteostracan agnathan *Norselaspis glacialis*^1^), and advanced technologies such as synchrotron imaging. Regarding early vertebrates, phase contrast X-ray synchrotron imaging of an acanthothoracid placoderm from the Early Devonian of Canadian Artic Archipelago has provided a detailed three-dimensional view of the skull vascularization and nerve canals^2^. The same technique also revealed vascular spaces in other taxa, such as the vascular mesh of the interolateral plate of the placoderm *Compagopiscis croucheri* (Late Devonian of Gogo Formation, Western Australia) or the vascular architecture of the humerus of *Eusthenopteron*, a sarcopterygian fish close to tetrapods (Late Devonian of Québec, Canada)^3^. Among tetrapods, traces of fossil blood vessels (arteries and veins) were reported for instance in the nasal bone of *Discosauriscus austriacus* from the Early Permian of Czech Republic^3^, in a *Triceratops* skull from the Upper Cretaceous of Hell Creek Formation, Montana^4^, in whale bones from the Middle-Upper Miocene of Peru)^5^, and in turtles from the Eocene of Germany and Miocene of Colombia^6^. These might represent internal moulds of the vascular walls or even the filling of the vascular channels^5^.

On the other hand, pulmonary vasculature (including traces such as internal moulds) has not been documented in vertebrate fossils to date. Pulmonary arteries and veins are documented in all groups of extant osteichthyans, except in Lepisosteidae (where the dorsal aorta serves as the afferent blood supply to the respiratory gas bladder), and in Chondrostei and Teleostei (which exhibit celiacomesenteric arteries)^7^. However, pulmonary arteries have never been properly described in any developmental stage of the extant coelacanth *Latimeria chalumnae.* This may be due to the challenge of visualizing vessels in x-ray microtomography (the unique effective non-invasive methodology to study all developmental stages of extant coelacanths) without the presence of a contrast solution or even to the reabsorption of vascularization in adult specimens of the extant coelacanth that possess a vestigial lung^8–10^. Based on histological thin sections of the lung lumen, pulmonary arteries were first erroneously described due to a misconception involving the pulmonary bony plates that are located on the vestigial lung^11,12^. More recently, these small and dense plates that surround the vestigial lung of *Latimeria chalumnae* were described as homologous to the calcified plates of Palaeozoic and Mesozoic coelacanth lungs^11^.

Here we describe, for the first time, the presence of pulmonary vessels in both fossil and extant coelacanths. These new observations confirm the air-breathing function of the so-called calcified organ in fossil coelacanths and the regressed state of the so-called vestigial lung in extant coelacanths. Presence of pulmonary vessels within this group resolves a piece of the puzzle regarding the evolution of air-breathing in vertebrates.

## Results

### Identification of pulmonary vasculature in the fossil coelacanth *Macropoma mantelli* (Late Cretaceous, Chalk Formation, Lewes, England)

**Figure 1:**
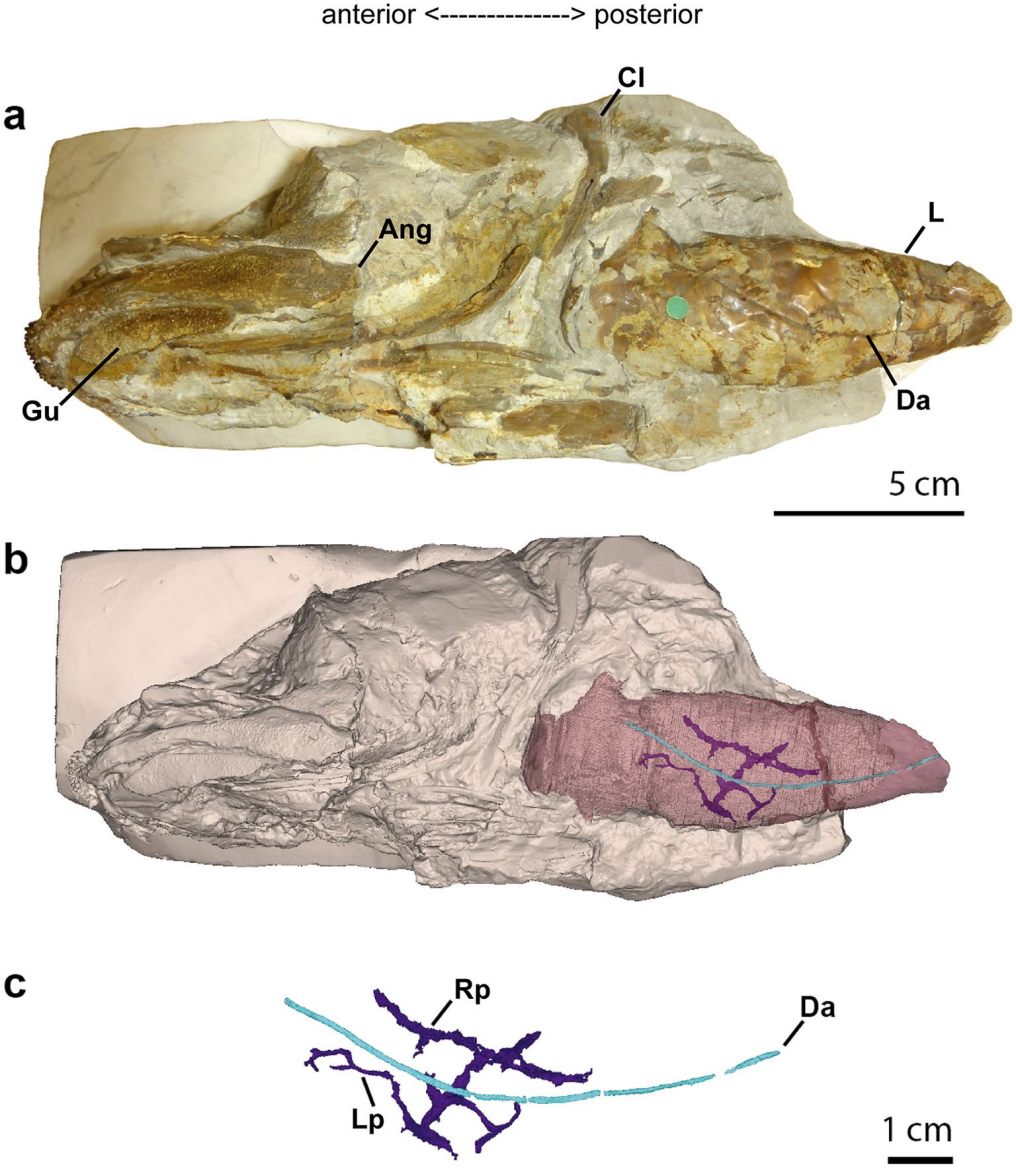
The pulmonary system of the Cretaceous coelacanth *Macropoma mantelli* NHMUK PV P 4270. (**a**) Photograph of specimen NHMUK PV 4270 in left lateral view. (**b**) Three-dimensional reconstruction of NHMUK PV 4270 with highlighting of the calcified lung, dorsal aorta and pulmonary arteries. (**c**) Close-up of the dorsal aorta and left and right pulmonary arteries. Pink, calcified lung; purple, pulmonary arteries; blue, dorsal aorta. *Ang* fragment of the angular bone, *Cl* cleithrum, *Da* dorsal aorta, *Gu* gular bone, *L* lung, *Lp* left pulmonary artery, *Rp* right pulmonary artery.

**Figure 2:**
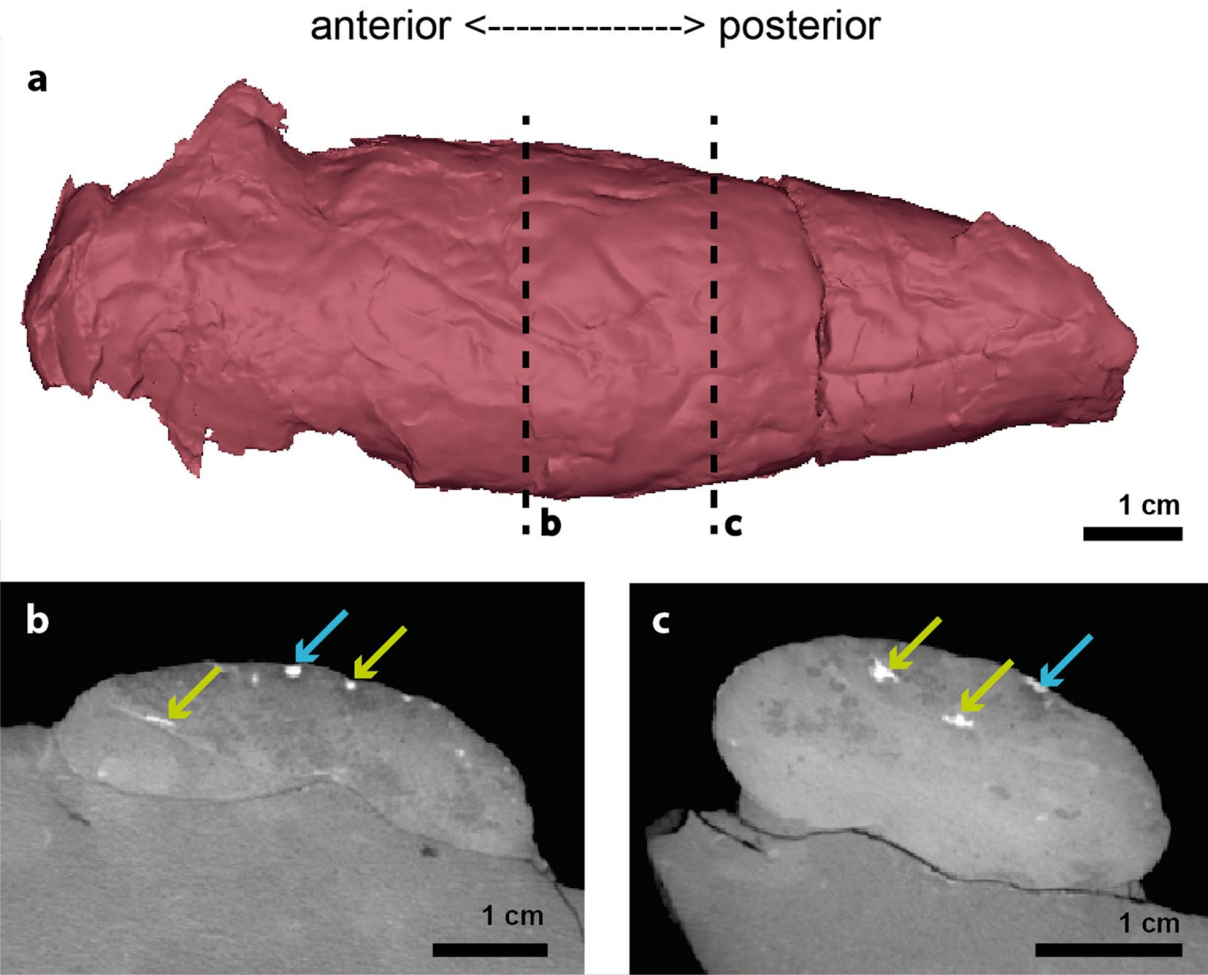
The calcified lung of *Macropoma mantelli* NHMUK PV P 4270. (**a**) Three-dimensional reconstruction of the calcified lung of the specimen. (**b**,**c**) Transverse sections of a high-resolution computerized axial tomography scan of NHMUK PV P 4270. Green arrows pointing to the pulmonary arteries internal to the lung lumen. Blue arrows, pointing to the dorsal aorta. Transverse tomography sections (**b**,**c**) corresponding to the successive dotted sections in (**a**).

The calcified lung of the specimen NHMUK PV 4270 is a well-developed unpaired organ, tubular in shape. It displays large superimposed bony plates surrounding a wide lumen (Fig. 1). Plates are rounded in shape and present a concave internal side. The lung is preserved in a ventral, but distorted, position, without division into chambers and without constriction (Fig. 1a), as known in some other fossil coelacanths. The anterior opening is not visible. The specimen, its tomographic segmentation, and three-dimensional reconstructions (Fig. 1a and b), clearly display the impression of the dorsal aorta, the most important vessel of the circulatory system, along the entire length of the external surface of the lung (Fig. 1a and b). Two pulmonary arteries, both with the same length, are also present parallel, but internal to the lung lumen and recovered by the bony plates (Figs. 1c, 2). These arteries are connected to each other by a transversal vessel. The pulmonary arteries give raise to branches of some arteries, as vascular channels (Fig. 1 c).

### Evidence of pulmonary vasculature in a juvenile extant coelacanth *Latimeria chalumnae*

**Figure 3:**
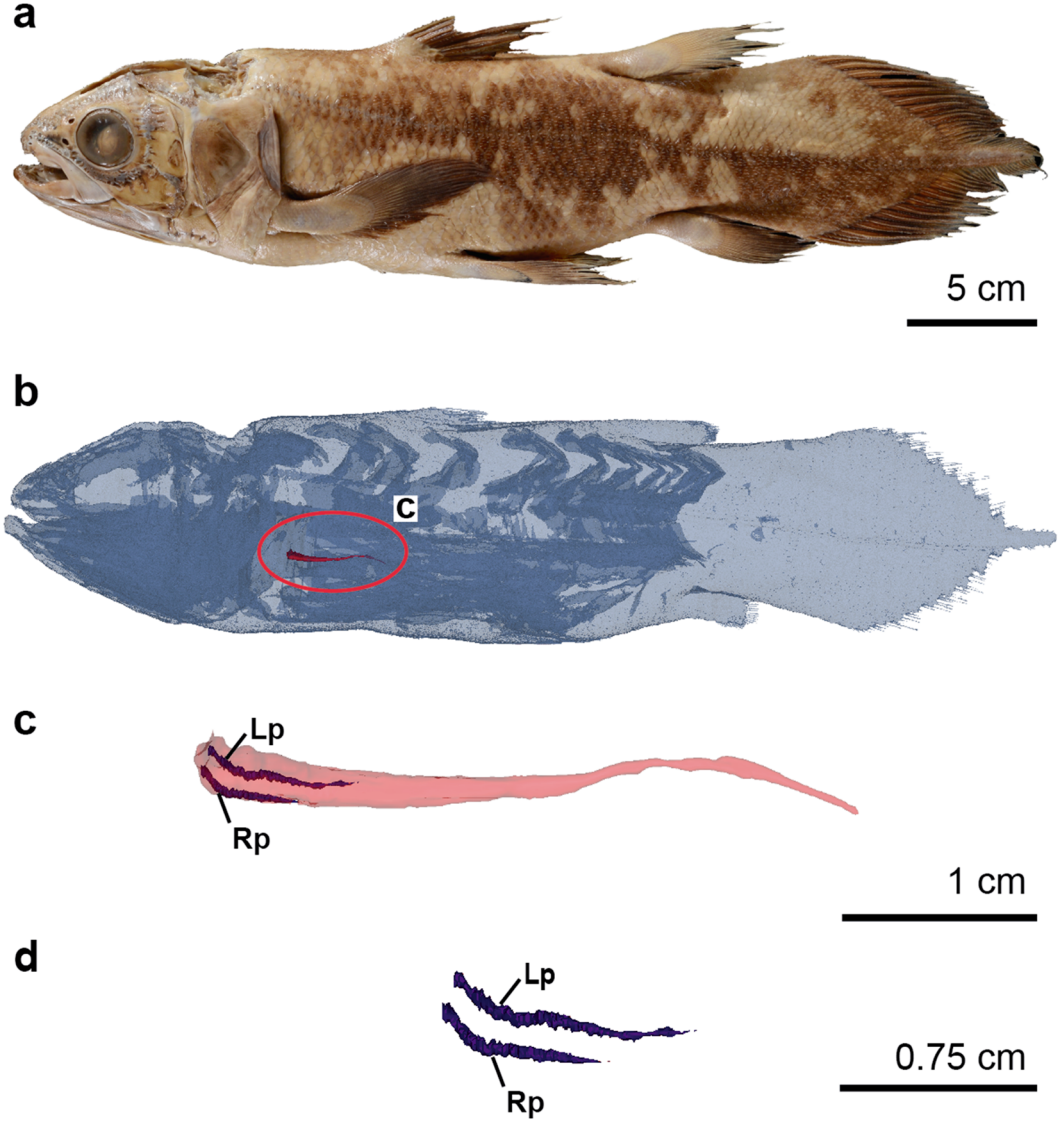
The pulmonary system of a juvenile specimen of the extant coelacanth *Latimeria chalumnae*. (**a**) Photograph of specimen CCC 94 (42.5 cm TL) in left lateral view. (**b**) Three-dimensional reconstruction of CCC 94. (**c**) Close-up of the three-dimensional reconstruction of the vestigial lung with left and right pulmonary arteries. (**d**) Close-up of the left and right pulmonary arteries. Pink, vestigial lung; purple, pulmonary arteries. *Lp* left pulmonary artery, *Rp* right pulmonary artery.

The lung of this juvenile coelacanth of 42.5 cm total length (specimen CCC 94) is unpaired, conical in shape and ventral to the oesophagus (Fig. 3). It is 2.157 cm long, corresponding to 15.88% of the length of the fatty organ and 5.13% of the length of the specimen (Fig. 3b). No bony plates are observed by X-ray tomography at this ontogenetic stage. The relative proportions between the lung and the total length (TL) of this specimen is lower in comparison to the same ration in embryos and higher in comparison with the same ratio in adult specimens^8^.

The specimen CCC 94 was historically injected with colloidal barite for observation of the circulatory system^12^, which reduced the quality of the tomography data for the soft tissues (Extended Data Fig. 1). However, this ancient injection has today enabled the visualization of the pulmonary arteries by X-ray tomography (usually not visible in coelacanth specimens without adding of colloidal barite) (Extended Data Fig. 1). The vestigial lung of CCC 94 is irrigated by two atrophied pulmonary arteries (Fig. 3c). Both pulmonary arteries run along the ventral surface of the vestigial lung. The dorsal aorta is not visible in this specimen, due to the artefacts generated by the massive injection of the contrast product. Indeed, barite strongly absorb X-rays, resulting in a local hyper-signal that obscures the anatomical structures on tomographic data. No vascularization canals or branches were observed within the fatty organ or in the connective tissue surrounding it (Fig. 3d).

## Discussion

Despite the rarity of preserved air-filled organs (AO^13^) in the fossil record, remains of pulmonary organs are known in an ornithuromorph bird, a salamander, a tadpole, as well as in almost all Palaeozoic and Mesozoic coelacanth families^10,11,14–18^. Almost all well-preserved fossil coelacanths, except some taxa such as *Diplurus*, *Coccoderma suevicum* and juveniles of *Axelrodichthys araripensis*, show well preserved ossified plates in their abdominal cavity^18^. These bony plates are superimposed, multilayered, and separated from one another by unossified connective tissue, likely enabling their mobility for volume adjustment during air-breathing, as well as providing protection against hydrostatic pressure^11,14^. The tubular structure, called the calcified organ, delimitated by a characteristic organization of these ossified plates has been recently considered as a structure constitutive of a functional lung in fossil coelacanths, homologous of the vestigial lung of extant coelacanths^8,11,14^.

The Cretaceous coelacanth specimen of *Macropoma mantelli* here studied has a well-developed pulmonary organ with ossified plates surrounding it, as well as well-developed dorsal aorta, pair of pulmonary arteries, and supplementary pulmonary branches irrigating this air-breathing organ. The extant coelacanth *Latimeria chalumnae*, which inhabits moderately deep waters and do not perform aerial gas exchange, shows a vestigial lung with vestigial vasculature, consisting of only a pair of pulmonary arteries.

Coelacanths are key taxa for the understanding of the evolutionary steps of the air-breathing history in osteichthyans. These occurrences of pulmonary vasculatures in both fossil and extant coelacanths reinforce the homology hypothesis between the fossil calcified organ and the vestigial lung. They then shed light on the evolution of the pulmonary complex within the actinistian clade and particularly on the loss of air-breathing during deep-marine water adaptation of the *Latimeria* relatives. Presence of lung vascular systems in both fossil and extant coelacanths also provides new anatomical elements concerning the evolutionary history of the vascular supply of air-filled organs in osteichthyans and the homology between lungs and gas bladders (also called swimbladders or air bladders).

Lungs and gas bladders are morphologically distinct air-filled organs that can serve the functions of buoyancy control and/or air-breathing. The homology (or lack thereof) between these organs has been a subject of discussion for almost 180 years^19,20^. Since the nineteenth century, this discussion has been based on morphological and molecular analysis. Among the molecular aspects, some authors have proposed homology based on the co-expression of genes in both tetrapod lungs and actinopterygian gas bladders^21^, homologous genes shared between gas bladders and the human lung^22^, and the similar surfactant system between both air-filled organs^23^. Morphologically, a homology was proposed based on different developmental stages of extant taxa that present a common origin from the posterior portion of the respiratory pharynx^24–27^), their identical arterial supply^7,13,25,28^, and their similar hydrostatic and/or respiratory function^13,29^. Indeed, the only morphological difference between lungs and gas bladders is the ventral and dorsal origins from the foregut^21,30^. Other characteristics previously used to differentiate both organs have been discarded in recent studies, as the lungs of actinopterygians and sarcopterygians, as well as the respiratory gas bladder of some actinopterygians, share certain morphological conditions^9,10^. However, sharing the same function and/or morphology does not necessarily imply homology^9,10,21,30^.

**Figure 4:**
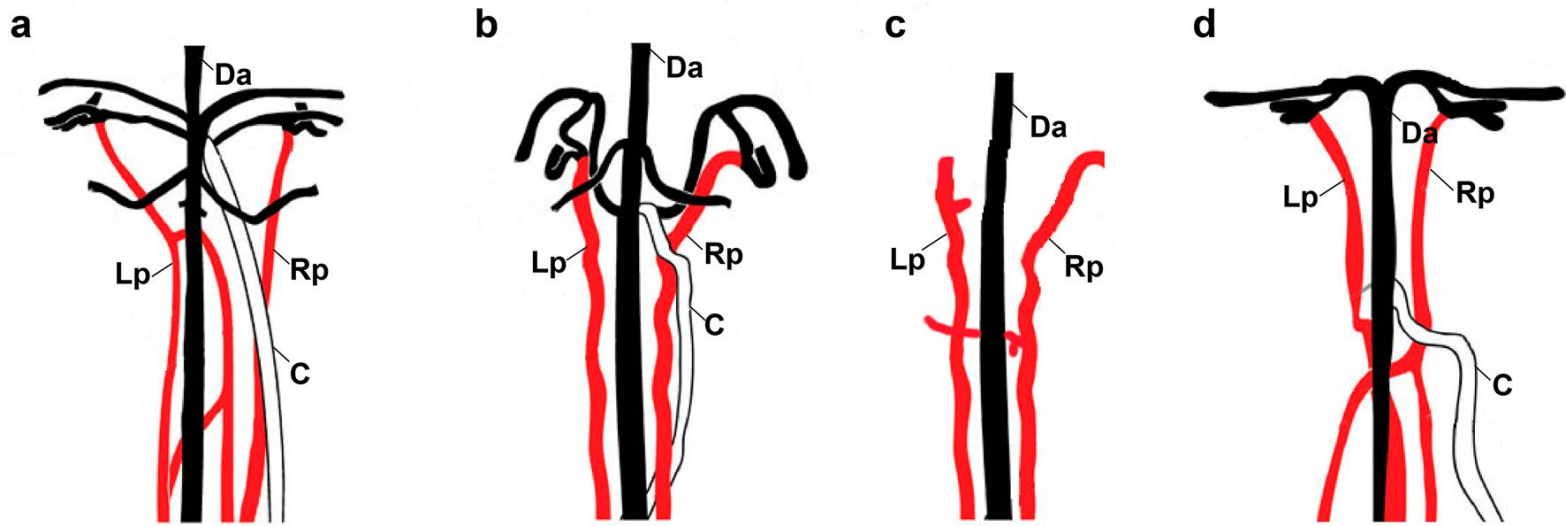
Schematic figure with a comparison of the pulmonary vascularization in Osteichthyes with potentially functional air-breathing organs (modified from Longo et al. 2013). (**a**) The actinopterygian *Polypterus senegalus*. (**b**) the holostean *Amia calva*. (**c**) the fossil coelacanth *Macropoma mantelli*. (**d**) the lungfish *Protopterus dolloi*. *C* celiacomesenteric artery, *Da* dorsal aorta, *Lp* left pulmonary artery, *Rp* right pulmonary artery.

**Figure 5:**
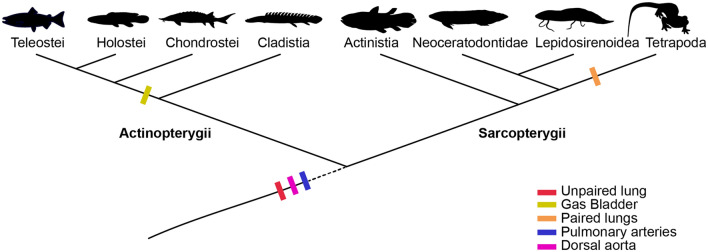
Schematic reconstruction of the evolutionary history of vertebrate lungs. Unpaired lung, pulmonary arteries and pulmonary arteries are plesiomorphies of Osteichthyes. Modified from Cupello et al.^10^. This figure was made with free silhouettes from PhyloPic.

Based on our results, we confirm that extant and fossil coelacanths possess pulmonary arteries homologous to the same paired branches of the air-filled organs (including gas bladders) of other osteichthyans (Fig. 4). We also support the hypothesis that pulmonary arteries are a synapomorphy of Osteichthyes (Fig. 5). If considering the common arterial supply as an indicator of homology between lungs and gas bladders, our results may contribute to a better understanding of the homology of air-breathing organs in vertebrates (Fig. 5), but further studies may explore better this condition, as well as the anatomy of fossil and extant chondrichthyans to support the absence of pulmonary arteries in this group.

## Methods

Specimen CCC 94 is a juvenile female of *Latimeria chalumnae* (42.5 cm TL) fished on a line in Comoro Islands in 1974^31^. This specimen was injected with colloidal barite to facilitate the observation of the circulatory system^12^. It was scanned with long propagation phase-contrast synchrotron X-ray microtomography at the ID19 beamline of the European Synchrotron Radiation Facility (Grenoble, France) in a plastic tube filled with water and with a propagation distance of 13 m. The beam produced by the wiggler was filtered by 2 mm of aluminium and 15 mm of copper, at a gap of 30 mm, and resulting in an average detected energy of 170 keV with a bandwidth of 85 keV FWHM. The voxel size of the scan is 28.43 μm and the final reconstructions of 85.29 μm was obtained after binning. Slices were reconstructed with filtered back-projection algorithm, a single distance phase-retrieval process^3,32^. Sub-scans were converted into 16-bit TIFF stacks and concatenated to generate a single complete scan. For more detail, see Cupello et al.^8^.

Specimen NHMUK PV 4270 is an adult specimen of the fossil coelacanth *Macropoma mantelli* from the Late Cretaceous of Chalk Formation, Lewes, Sussex, UK. X-ray computed micro-tomography (µCT) scanning of this specimen was performed at the AST-RX Platform of the Muséum national d’Histoire naturelle, Paris, France. The voltage was 140 kV, current 350 mA, voxel size 127.85 µm and the view number was 1500.

For both specimens, segmentation and three-dimensional rendering were realized using the software MIMICS Innovation Suite 20.0 to 25.0 (Materialise) at the Laboratório de Ictiologia Tempo e Espaço of the Universidade do Estado do Rio de Janeiro.

### Supplementary Information

Supplementary Information 1.

Supplementary Information 2.

Supplementary Information 3.

Supplementary Information 4.

## Data Availability

Data can be made available by the corresponding author upon request.
