# Supplementary material for: Pulmonary arteries in coelacanths shed light on the vasculature evolution of air-breathing organs in vertebrates
Source: Sci Rep. 2024 May 9;14:10624. doi: 10.1038/s41598-024-61065-8 (PMC11082188; doi:10.1038/s41598-024-61065-8)
Supplement: Supplementary file 3 — Supplementary Information 3. [file 41598_2024_61065_MOESM3_ESM.docx]

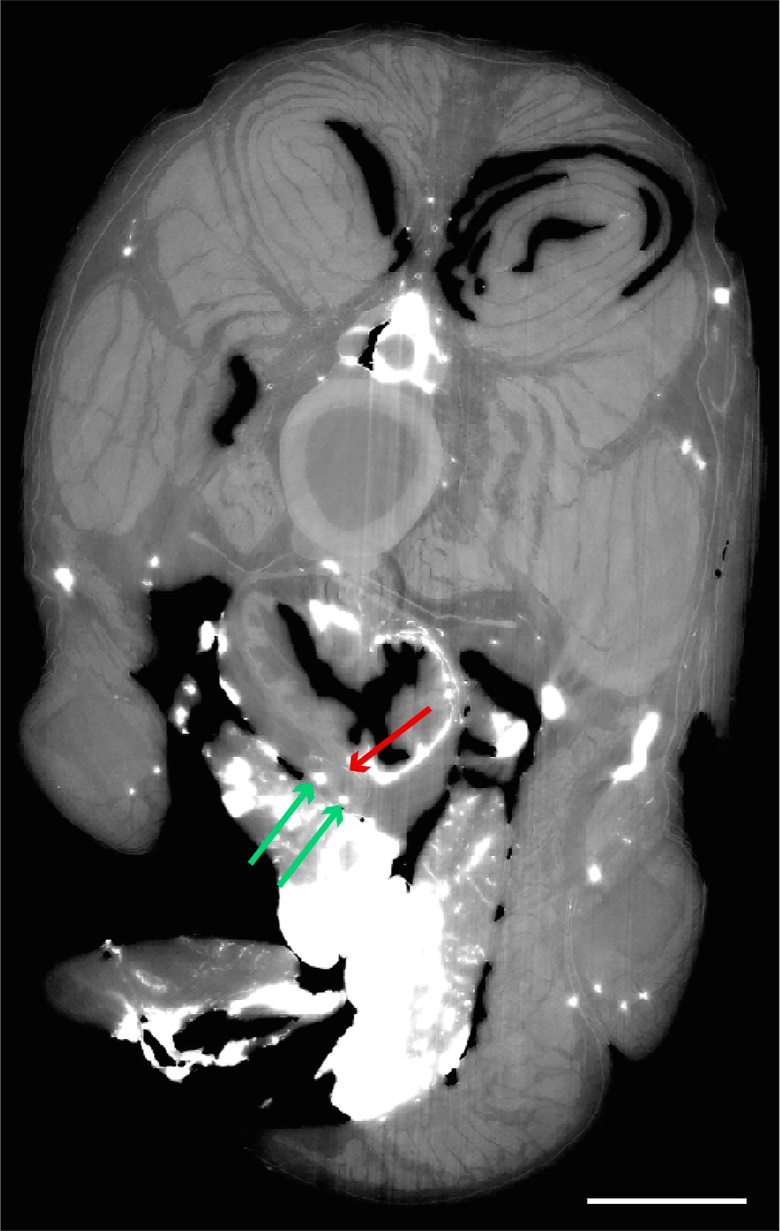


Extended Data figure 1: Section of a long propagation phase-contrast synchrotron X-ray microtomography scan of the juvenile of *Latimeria chalumnae* (specimen CCC 94). Red arrow pointing to the lung boundary. Green arrows pointing to the pulmonary arteries internal to the lung lumen. Scale bar, 1 cm.
