# Supplementary material for: Pulmonary arteries in coelacanths shed light on the vasculature evolution of air-breathing organs in vertebrates
Source: Sci Rep. 2024 May 9;14:10624. doi: 10.1038/s41598-024-61065-8 (PMC11082188; doi:10.1038/s41598-024-61065-8)
Supplement: Supplementary file 4 — Supplementary Information 4. [file 41598_2024_61065_MOESM4_ESM.docx]

**How to enable playing of 3D content in PDFs** ([Enable 3D content in PDF, Adobe Acrobat and Reader](https://helpx.adobe.com/acrobat/using/enable-3d-content-pdf.html)):

1. When you open a PDF with 3D content, you will be prompted to enable the feature if you trust the document.
2. Select "Options" and then choose one of the following options:

- Trust this document one time only.
- Trust this document always.

1. Double-click on the blank page.
2. On the right side of the page, you will find:

- Tree

- Options

- Root

- Parts

5- All 3D models are included within this file.
