# Supplementary material for: Pulmonary arteries in coelacanths shed light on the vasculature evolution of air-breathing organs in vertebrates
Source: Sci Rep. 2024 May 9;14:10624. doi: 10.1038/s41598-024-61065-8 (PMC11082188; doi:10.1038/s41598-024-61065-8)
Supplement: Supplementary file 1 — Supplementary Information 1. [file 41598_2024_61065_MOESM1_ESM.pdf]

materialise mimics
innovation suite

Rotate | Zoom | Pan | Full Screen Mode : CTRL + L
